# The systematic review and meta-analysis evaluated the efficacy and safety of nefopam for catheter-related bladder discomfort based on randomized controlled trials

**DOI:** 10.3389/fphar.2023.1305844

**Published:** 2023-11-24

**Authors:** Junpeng Chi, Jinhui Wu, Keyuan Lou, Jian Ma, Jitao Wu, Yuanshan Cui

**Affiliations:** ^1^ Department of Urology, The Affiliated Yantai Yuhuangding Hospital of Qingdao University, Yantai, China; ^2^ Department of Gastrointestinal Surgery Ward II, The Affiliated Yantai Yuhuangding Hospital of Qingdao University, Yantai, China

**Keywords:** catheter-related bladder discomfort, randomized controlled trials, meta-analysis, CRBD, nefopam

## Abstract

**Background:** Catheter-related bladder discomfort (CRBD) is a frequent occurrence following urinary catheterization during surgical procedures, as well as a commonly experienced bladder pain syndrome after surgery. There have been various studies on drugs and interventions to manage CRBD, but their comparative efficacy and safety are still a topic of debate. We conducted a meta-analysis to assess the efficacy and safety of nefopam for managing postoperative CRBD.

**Methods:** A systematic search of PubMed, Embase, Cochrane Library, and Web of Science was conducted to find randomized controlled trials (RCTs) on using nefopam in postoperative CRBD. The study employed the Preferred Reporting Items for Systematic Reviews and Meta-Analyses. Data analysis was performed using RevMan version 5.4.1.

**Results:** Five RCTs with 405 patients were analyzed to evaluate the efficacy of nefopam on postoperative CRBD. Short-term and long-term periods were defined as within 6 h and longer than 12 h after surgery, respectively. The incidence and severity of CRBD were compared between the two groups during these time periods. The analysis proved that nefopam reduced the short-term incidence of postoperative CRBD (RR 0.36; 95% CI, 0.18–0.70; *p* = 0.003, I^2^ = 78%) and the long-term incidence (RR 0.49; 95% CI, 0.32–0.74; *p* = 0.0007, I^2^ = 0%) significantly. We compared the incidence of moderate-to-severe CRBD between groups based on the scaling system (none, mild, moderate, and severe). This was used to assess the severity of postoperative CRBD. The results showed that patients in the nefopam group had a significantly lower incidence of moderate-to-severe CRBD compared to those in the placebo group in the short-term (RR 0.19; 95% CI, 0.10–0.34; *p* < 0.00001; I^2^ = 0%). However, there were no significant differences between the two groups in the incidence of moderate-to-severe CRBD in the long-term (RR 0.61; 95% CI, 0.21–1.76; *p* = 0.36; I^2^ = 0%). There were no significant variations in the occurrence of adverse events between the nefopam and control groups, mainly including postoperative nausea and vomiting (PONV) (RR 1.14; 95% CI, 0.40–3.21; *p* = 0.81), and tachycardia (RR 0.25; 95% CI, 0.03–2.11, *p* = 0.20).

**Conclusion:** The findings of this meta-analysis indicate that nefopam significantly reduced the incidence of short or long-term postoperative CRBD. Nefopam decreased the severity of postoperative CRBD, particularly significantly reducing the occurrence of moderate to severe CRBD in the short-term. Overall, patients have good tolerance and no apparent side effects.

**Systematic Review Registration**: identifier PROSPERO (CRD42023475012)

## 1 Introduction

Large-diameter urinary catheters used after surgical procedures, particularly urological surgeries, may lead to CRBD. This discomfort is characterized by a sense of urgency and increased frequency in urination, accompanied or not by involuntary loss of urine (Bai et al., 2015). CRBD symptoms resemble those of an overactive bladder (OAB) (Yoshimura and Chancellor, 2002). This discomfort, not only impairs postoperative recovery but also contributes to patient dissatisfaction and prolonged hospital stays. Managing CRBD effectively is crucial not only for improving patient outcomes but also for optimizing healthcare resources.

The current treatment options for postoperative CRBD are diverse, and novel strategies are continually sought to address this issue. Various agents have been studied and verified for the prevention and treatment of CRBD. Some of these agents include ketamine, dexmedetomidine, antimuscarinics, gabapentin, tolterodine, and tramadol (Shi et al., 2021; Zhou et al., 2021; Li et al., 2022; Lu et al., 2022). Recently, scholars have suggested that nefopam may be effective in managing CRBD (Charoenpol et al., 2023; Ren et al., 2023). Nefopam, a centrally acting analgesic with anticholinergic properties, shows potential as an intervention for mitigating postoperative bladder discomfort (Evans et al., 2008). However, there was no comprehensive meta-analysis to assess the efficacy and safety of nefopam in CRBD.

This essay conducts a systematic review and meta-analysis of randomized controlled trials (RCTs) to assess the efficacy and safety of nefopam in managing this condition in postoperative urosurgical patients. The objective of this essay is to synthesize the findings of existing RCTs to provide a rigorous assessment of nefopam’s role in addressing CRBD, with implications for urosurgical practice.

## 2 Materials and methods

### 2.1 Search strategy

Up to 1 September 2023, we conducted a systematic literature search using several electronic databases, including PubMed, Embase, Cochrane Library, and Web of Science. The search terms employed were a combination of controlled vocabulary terms (e.g., MeSH terms) and free-text keywords. We included RCTs that investigated the efficacy and safety of nefopam in managing CRBD in urosurgical patients. The following search terms were applied for the search: RCT, nefopam, and CRBD.

### 2.2 Inclusion and exclusion criteria

In our systematic review and meta-analysis, we followed a rigorous set of inclusion and exclusion criteria to select relevant studies for analysis. Two authors utilized PICOS (Patient, Intervention, Control, Outcome, Study design) criteria to include relevant RCTs. All authors independently browsed and read all searched articles, and the final list of included articles was decided through a consensus discussion.

#### 2.2.1 Inclusion criteria

1) Participants: Studies involving adult urosurgical patients who experienced CRBD were eligible for inclusion. 2) Intervention: Included studies administered nefopam as an intervention to manage CRBD. 3) Control: The included studies used saline as a placebo control treatment for managing CRBD. 4) Outcome Measures: Studies reporting relevant outcome measures, including the incidence of CRBD, the severity of CRBD symptoms, and postoperative side effects were considered. 5) Study Design: We included RCTs that investigated the efficacy and safety of nefopam in managing CRBD in urosurgical patients.

#### 2.2.2 Exclusion criteria

**FIGURE 1:**
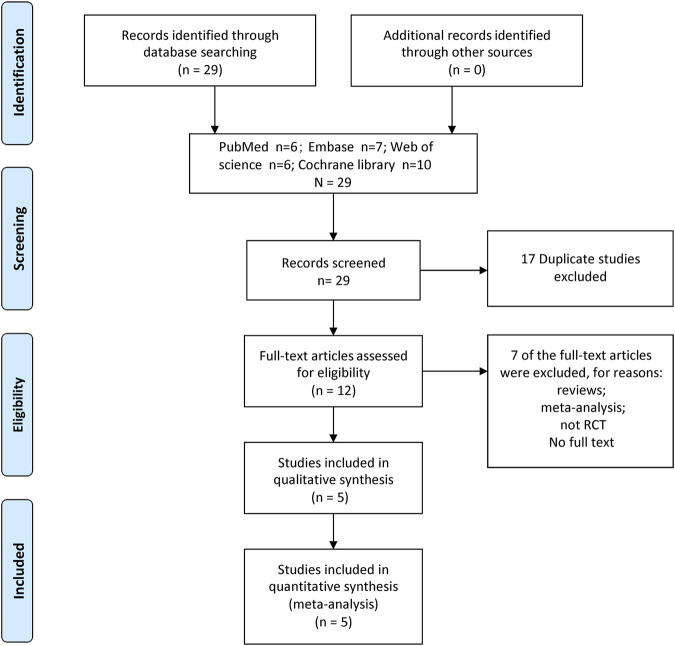
A flow diagram of the study selection process.

Review, meta-analysis, none RCT, and no full text were not considered. Figure 1 depicts the PRISMA flow diagram.

### 2.3 Data extraction

The retrieved studies were screened by two independent reviewers using predefined inclusion and exclusion criteria. Disagreements were resolved through discussion and consensus. A standardized data extraction form was utilized to collect the following information from each included study: 1) study characteristics (e.g., authors, publication year, country); 2) therapy in experimental group; 3) therapy in control group; 4) participant quantity demographics; 5) catheter size; 6) inflated balloon and volume; 7) types of surgery; 8) primary outcomes; 9) secondary outcomes; (10) check points and time. Table 1 showed the **c**haracteristics of included studies.

**TABLE 1:** Basic characteristics of included studies.

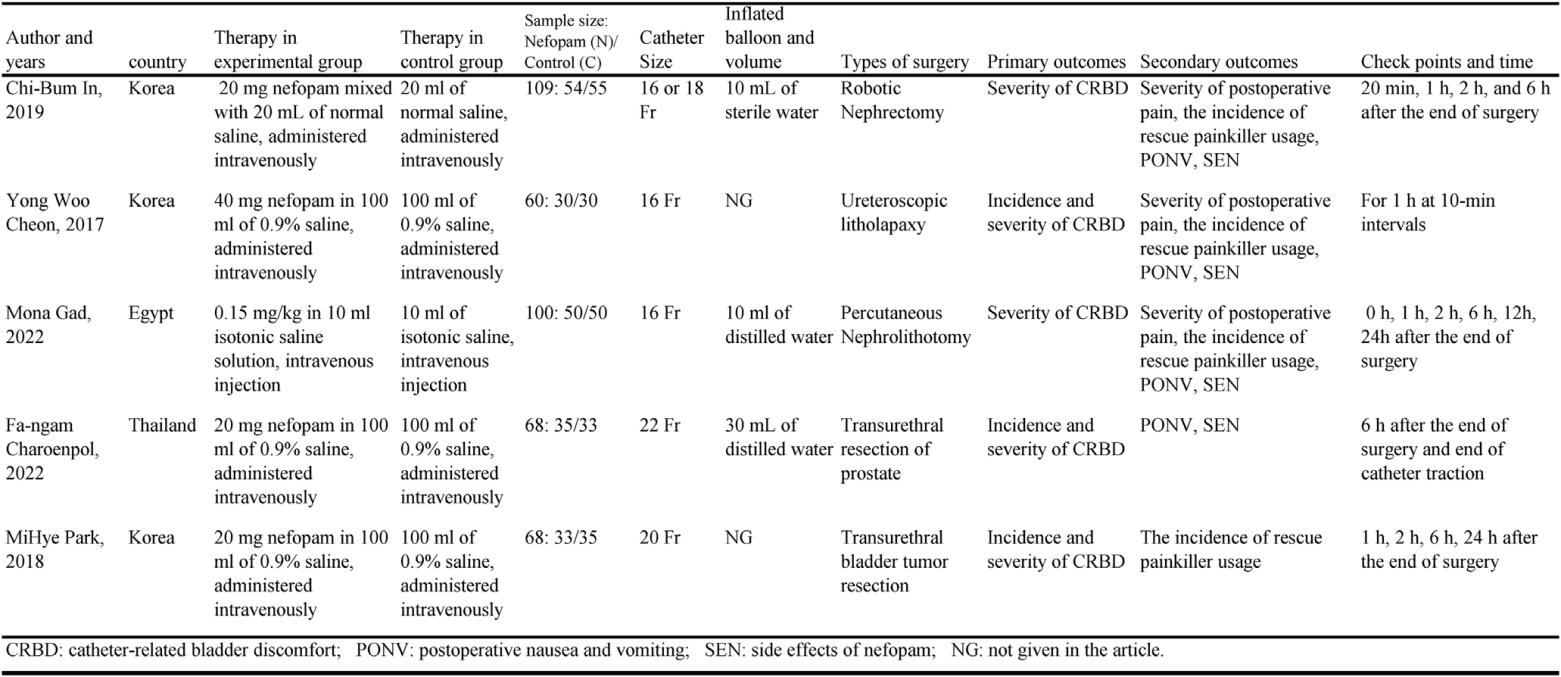

### 2.4 Quality assessment

**FIGURE 2:**
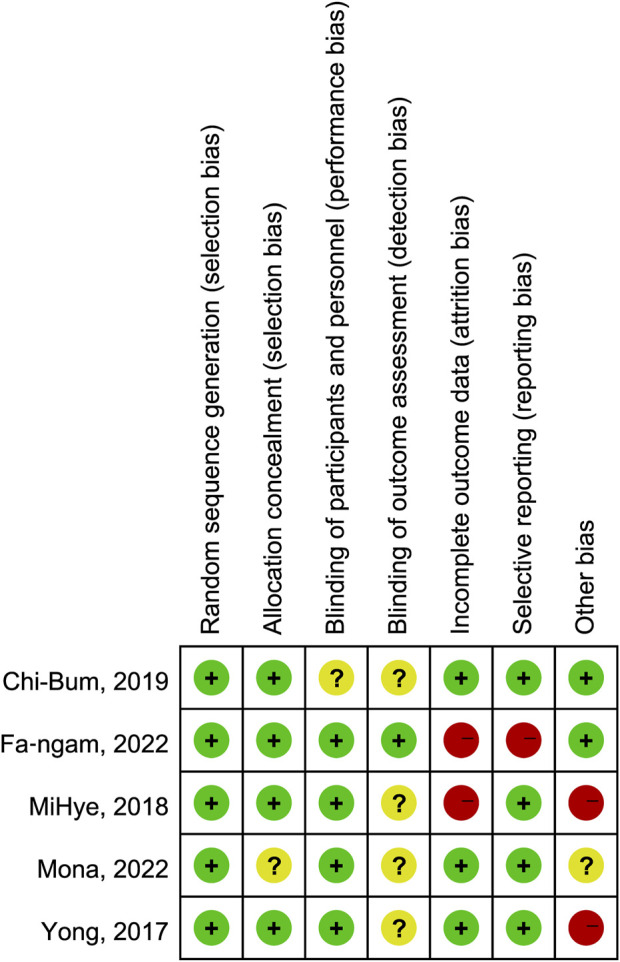
Risk of bias summary of the included studies.

**FIGURE 3:**
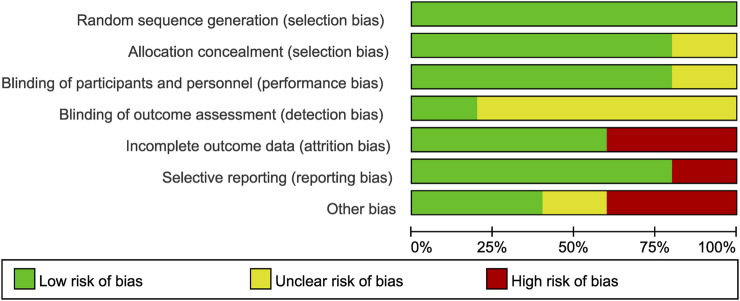
Risk of bias graph of the included studies.

To evaluate the quality of the included studies, we employed the Cochrane Risk of Bias tool. (Higgins et al., 2011). This tool assesses the risk of bias in several domains, including random sequence generation, allocation concealment, blinding of participants and personnel, blinding of outcome assessment, incomplete outcome data, selective reporting, and other potential sources of bias. Each domain was assessed as “low risk,” “high risk,” or “unclear risk” of bias. Figure 2 and Figure 3 demonstrated an overview of the risk of bias. The Supplementary Material S1–S3 entail funnel plots included in the study.

### 2.5 Statistical analysis

A meta-analysis was conducted using Review Manager software version 5.4.1 (Cochrane Collaboration, Oxford, United Kingdom). Only variables evaluated by at least two studies were pooled. The mean difference (MD) with 95% confidence intervals (CIs) was used for continuous data, while risk ratios (RRs) with 95% confidence intervals were used for dichotomous data. When there was no significant heterogeneity (I^2^ not greater than 50%), a fixed-effect model was employed; otherwise (I^2^ greater than 50%), a random-effects model was used. Statistical significance was determined using a *p*-value of less than 0.05.

## 3 Results

We identified 29 relevant studies through a comprehensive search process. After thoroughly examining the complete texts of these studies, we excluded 24 of them based on the reasons provided in Figure 1. Finally, the meta-analysis included five RCTs with 405 patients (202 in the nefopam group and 203 in the control group) (Cheon et al., 2018; Park et al., 2018; In et al., 2019; Gad et al., 2022; Charoenpol et al., 2023).

### 3.1 Characteristics of included studies

Five studies, all of which were RCTs, were analyzed. The studies had adequate participant numbers for analysis. Each study used a computer-generated block randomization list to assign participants to either the experimental or control group. The five studies focused on different common surgical procedures in urology.

### 3.2 Efficacy of nefopam on postoperative CRBD

The primary outcome of our meta-analysis was the assessment of nefopam’s efficacy in managing CRBD in urosurgical patients. This mainly includes the effects of nefopam on the incidence and severity of postoperative CRBD.

#### 3.2.1 Incidence of postoperative CRBD

We compared the incidence rates of postoperative CRBD between the short-term and long-term groups according to the timing of CRBD occurrence (we define short-term as within 6 hours after surgery and long-term as more than 12 h after surgery), and data are presented as numbers. Three studies enrolling 196 participants (98 in the nefopam group and 98 in the control group) (Cheon et al., 2018; Park et al., 2018; Charoenpol et al., 2023) were used to analyze the impact of nefopam on the incidence of postoperative CRBD.

**FIGURE 4:**
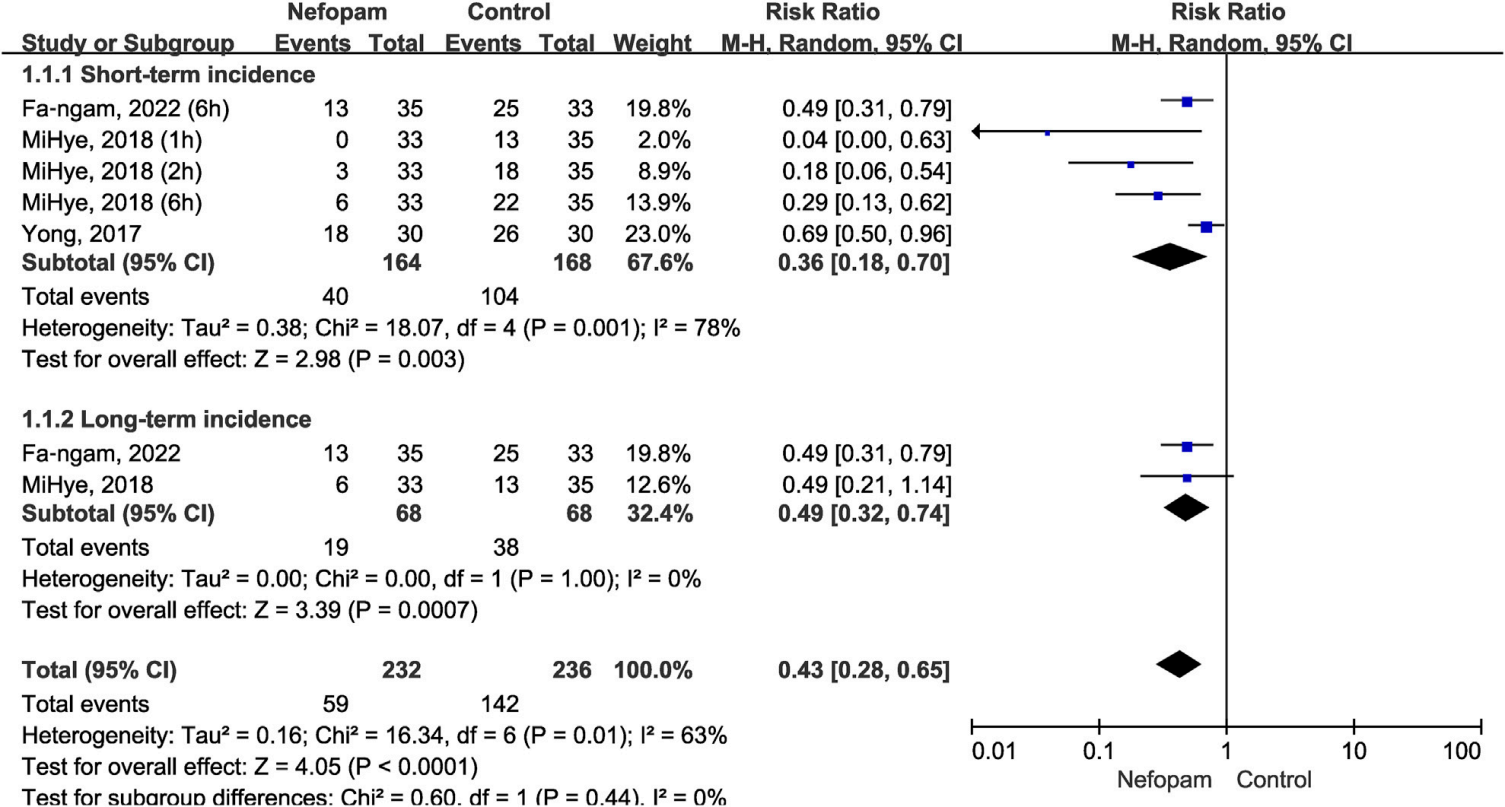
Incidence of catheter-related bladder discomfort in nefopam vs. placebo.

Nefopam reduced the short-term incidence of postoperative CRBD (RR 0.36; 95% CI, 0.18–0.70; *p* = 0.003, I^2^ = 78%) significantly; meanwhile, nefopam reduced the long-term incidence of postoperative CRBD (RR 0.49; 95% CI, 0.32–0.74; *p* = 0.0007, I2 = 0%). Overall, the meta-analysis revealed a statistically significant reduction in the incidence rates of postoperative CRBD favoring nefopam treatment (RR 0.43; 95% CI, 0.28–0.65; *p* < 0.0001, I^2^ = 63%) (Figure 4).

#### 3.2.2 The severity of postoperative CRBD

Similarly, according to the short-term and long-term grouping, we compared the incidence of moderate-to-severe CRBD between groups based on the scaling system (none, mild, moderate, and severe). The data is presented in numerical form to assess the severity of postoperative CRBD. Three studies enrolling 196 participants (98 in the nefopam group and 98 in the control group) (Cheon et al., 2018; Park et al., 2018; Charoenpol et al., 2023) were used to analyze the impact of nefopam on the severity of postoperative CRBD.

**FIGURE 5:**
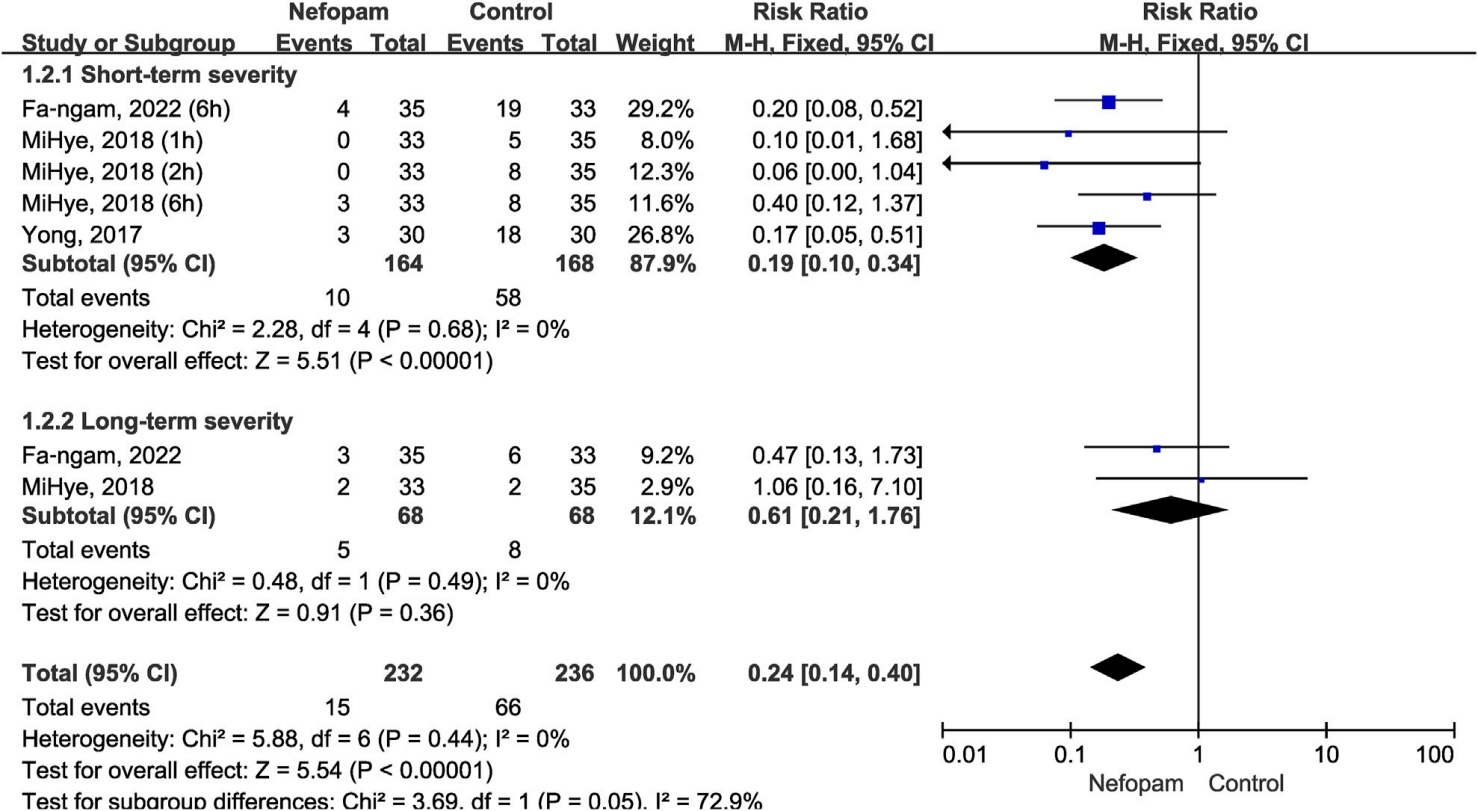
Incidence of moderate-to-severe catheter-related bladder discomfort in nefopam vs. placebo.

Patients in the nefopam group showed a significantly lower severity of CRBD than those in the placebo group in the short-term (RR 0.19; 95% CI, 0.10–0.34; *p* < 0.00001; I^2^ = 0%). In contrast, there were no meaningful differences between the two groups in the severity of CRBD in the long-term (RR 0.61; 95% CI, 0.21–1.76; *p* = 0.36; I^2^ = 0%). Overall, the meta-analysis revealed a statistically significant reduction in the severity of postoperative CRBD favoring nefopam treatment (RR 0.24; 95% CI, 0.14–0.40; *p* < 0.00001) (Figure 5).

### 3.3 Safety of postoperative nefopam

In addition to assessing efficacy, we also examined the safety profile of nefopam in the included RCTs. We evaluated the incidence of adverse events and patients tolerance to nefopam to determine its safety. The 202 patients in the nefopam group included in the five articles did not experience any specific adverse drug reactions, and overall, the patients exhibited good tolerance.

**FIGURE 6:**
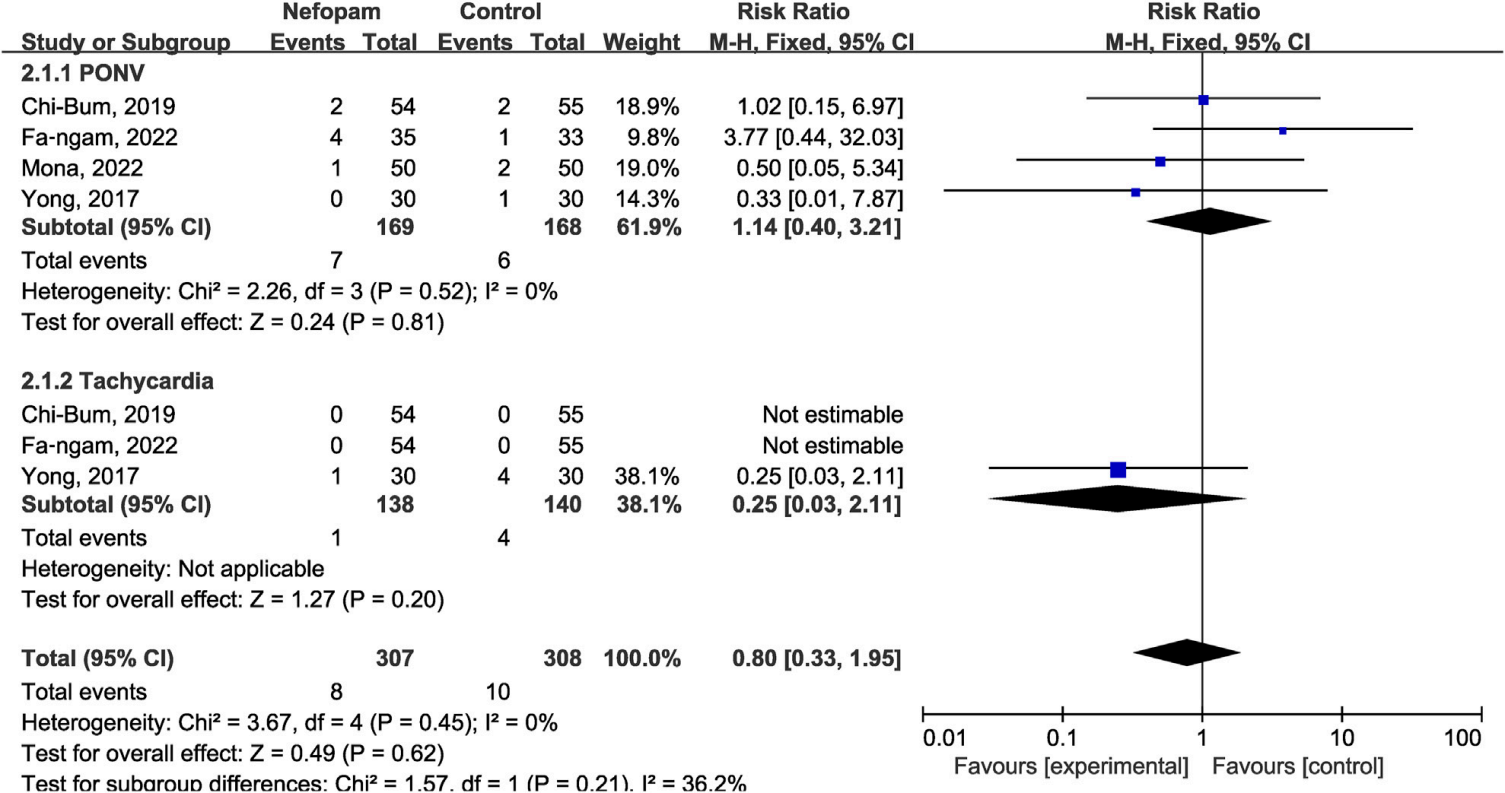
Forest plot of postoperative side effects.

There were no significant variations in the occurrence of adverse events between the nefopam and control groups, mainly including postoperative nausea and vomiting (PONV) (RR 1.14; 95% CI, 0.40–3.21; *p* = 0.81), and tachycardia (RR 0.25; 95% CI, 0.03–2.11; *p* = 0.20) (Figure 6).

## 4 Discussion

CRBD is a significant and distressing phenomenon in medical practice, posing a unique challenge to healthcare providers in ensuring patient comfort and wellbeing. Despite its widespread occurrence, effective management of CRBD remains a complex and underexplored territory. Existing treatment options, including antispasmodic agents and analgesics, often provide suboptimal relief and may carry adverse effects (Wilson, 2008; Bach et al., 2020; Lin et al., 2021). In this context, nefopam, a non-opioid analgesic with a unique pharmacological profile, has emerged as a potential candidate for CRBD management (Evans et al., 2008).

CRBD is caused by involuntary contractions of the bladder, mediated by muscarinic receptors located in the urothelium and on efferent nerves (Andersson and Wein, 2004). Catheters cause bladder wall stretching and mechanical irritation, resulting in urgency, discomfort, and pain. They can also trigger a local inflammatory response and the release of inflammatory mediators, contributing to discomfort and urgency. Catheter-induced irritation can lead to involuntary contractions, worsening symptoms. Patients with this condition often experience psychological discomfort, which involves neural pathways transmitting signals of discomfort and urgency to the central nervous system (Li et al., 2014; Janssen et al., 2017). Current treatment options, such as antimuscarinic drugs, offer limited relief and may be associated with undesirable side effects (Bai et al., 2015; Jang et al., 2020; Li et al., 2022). Nefopam is a nonopioid analgesic with a unique chemical structure. Unlike traditional opioids, nefopam does not bind to opioid receptors (Heel et al., 1980).

In clinical studies conducted in various medical contexts, nefopam has demonstrated a generally favorable adverse event profile. Common side effects include nausea, dizziness, and dry mouth (Chanques et al., 2011). However, these side effects are typically mild to moderate in severity. One of the notable advantages of nefopam is its lack of opioid-related side effects, such as respiratory depression, gastrointestinal events, headache, pruritus, tolerance, and dependence (Smith, 2009; Funk et al., 2014; Els et al., 2017). This characteristic is especially valuable for patients who may be at risk of these issues or those requiring non-opioid alternatives.

While preliminary findings indicate promise, it is vital to acknowledge the need for more robust, multicenter clinical trials to confirm nefopam’s efficacy in CRBD and further delineate its role in clinical practice. Nefopam primarily acts as a s Serotonin-Norepinephrine-Dopamine Reuptake Inhibitor (SNDRI) (Fuller and Snoddy, 1993; Urwin and Smith, 1999). By inhibiting the reuptake of these neurotransmitters, nefopam modulates pain perception and transmission in the central nervous system. This mechanism may be advantageous in CRBD, where neural pathways play a role in symptom generation. Nefopam may modulate neural pathways involved in CRBD, potentially reducing the transmission of discomfort signals to the central nervous system (Czuczwar et al., 2011). Nefopam exhibits smooth muscle relaxation properties, making it an antispasmodic agent. In the context of CRBD, where detrusor muscle spasms contribute to discomfort, nefopam’s muscle-relaxant effect is of particular interest. Nefopam’s ability to modulate pain perception could alleviate the discomfort associated with CRBD, improving the patient’s overall experience (Urwin and Smith, 1999; Dacero, 2004).

We noticed that three out of the five included studies used a dose of 20 mg of nefopam, which is consistent with the analgesic dose commonly used in our clinical practice. Previous studies have shown 20 mg of nefopam is approximately equivalent to 12 mg of morphine in terms of pain relief, but with reduced side effects (Sunshine and Laska, 1975). In Yong Woo’s study, no significant differences were found between groups in terms of intraoperative hypertension, tachycardia, and postoperative side effects such as PONV, somnolence, hyperhidrosis, headache, and blurred vision (Cheon et al., 2018). Adverse effects reported in Fa-Ngam’s study included nausea (8.57%), vomiting (2.86%), cold sweating (2.86%), and dry mouth (2.86%) in the nefopam group. However, there were no significant differences with control group (Charoenpol et al., 2023).

Nefopam can be delivered orally, intravenously, and through muscle injection, providing flexibility in tailoring treatment to individual patient needs. The dosage and duration of treatment can be adjusted based on the severity of postoperative CRBD and the patient’s overall response to the medication. Several studies have been conducted to evaluate the effectiveness of nefopam in alleviating postoperative catheter-related bladder discomfort. These studies have shown promising results, indicating that nefopam can significantly reduce the intensity and frequency of bladder discomfort, improve patient satisfaction, and enhance overall postoperative recovery (Cheon et al., 2018; Park et al., 2018; In et al., 2019; Gad et al., 2022; Charoenpol et al., 2023). This is also in line with the philosophy of Enhanced Recovery After Surgery (ERAS). Nefopam shows potential as a treatment for postoperative catheter-related bladder discomfort. Further research is needed to determine its effectiveness, proper dosage, and long-term safety in this context.

We performed this meta-analysis with a total of five articles, and our study proved that nefopam significantly decreased the incidence of CRBD and the severity of CRBD in patients with urinary catheters, especially in the short-term postoperative period. We also noticed that nefopam did not significantly reduce the incidence of moderate-to-severe CRBD in patients with long-term postoperative indwelling urinary catheters. This may be attributed to the limited number of study included in our review, and it is also possible that the degree of pain and discomfort decreases as the duration of indwelling catheter increases. Additionally, the duration of indwelling catheter in patients undergoing nephrectomy and ureteroscopic litholapaxy is shorter compared to bladder tumor resection and prostatectomy. These factors combined resulted in a statistically insignificant impact of nefopam on the incidence of moderate-to-severe CRBD in patients with long-term postoperative indwelling urinary catheters.

The studies analyzed were all RCTs, which improved the reliability of the findings. Despite the overall high quality of the included studies, our analysis did have certain limitations. Firstly, this meta-analysis only included 5 studies with small sample size, which was limited to the number of relevant original studies. Secondly, the patient’s perioperative indicators may be inconsistent, including different sizes of catheter, different doses of nefopam, different timing of administration, and various types of surgeries. In addition, due to the presence of heterogeneity, this may lead to a certain publication bias. This meta-analysis is important for evaluating the efficacy of nefopam compared to placebo in preventing or treating symptoms of CRBD when considering heterogeneity among articles.

Nefopam, as the first study conducted on the topic, has been found to be more efficacy than placebo in relieving symptoms of CRBD. Extensive data analysis supports the conclusion that nefopam significantly outperforms placebo in alleviating CRBD symptoms. In the future, we look forward to more clinical scientists focusing on the plasma concentration of nefopam and hope for more clinical research data to supplement and further improve our analysis in the article’s main subject.

## 5 Conclusion

The meta-analysis demonstrates that nefopam administration mitigates the frequency and severity of early postoperative CRBD without causing evident side effects. Further investigations, including well-designed RCTs with standardized dosing regimens and assessment methods, are needed to refine the efficacy and safety of nefopam in CRBD management.

## Data Availability

The original contributions presented in the study are included in the article/Supplementary Material, further inquiries can be directed to the corresponding authors.
