# Supplementary figures and images for: The systematic review and meta-analysis evaluated the efficacy and safety of nefopam for catheter-related bladder discomfort based on randomized controlled trials
Source: Front Pharmacol. 2023 Nov 24;14:1305844. doi: 10.3389/fphar.2023.1305844 (PMC10704467; doi:10.3389/fphar.2023.1305844)

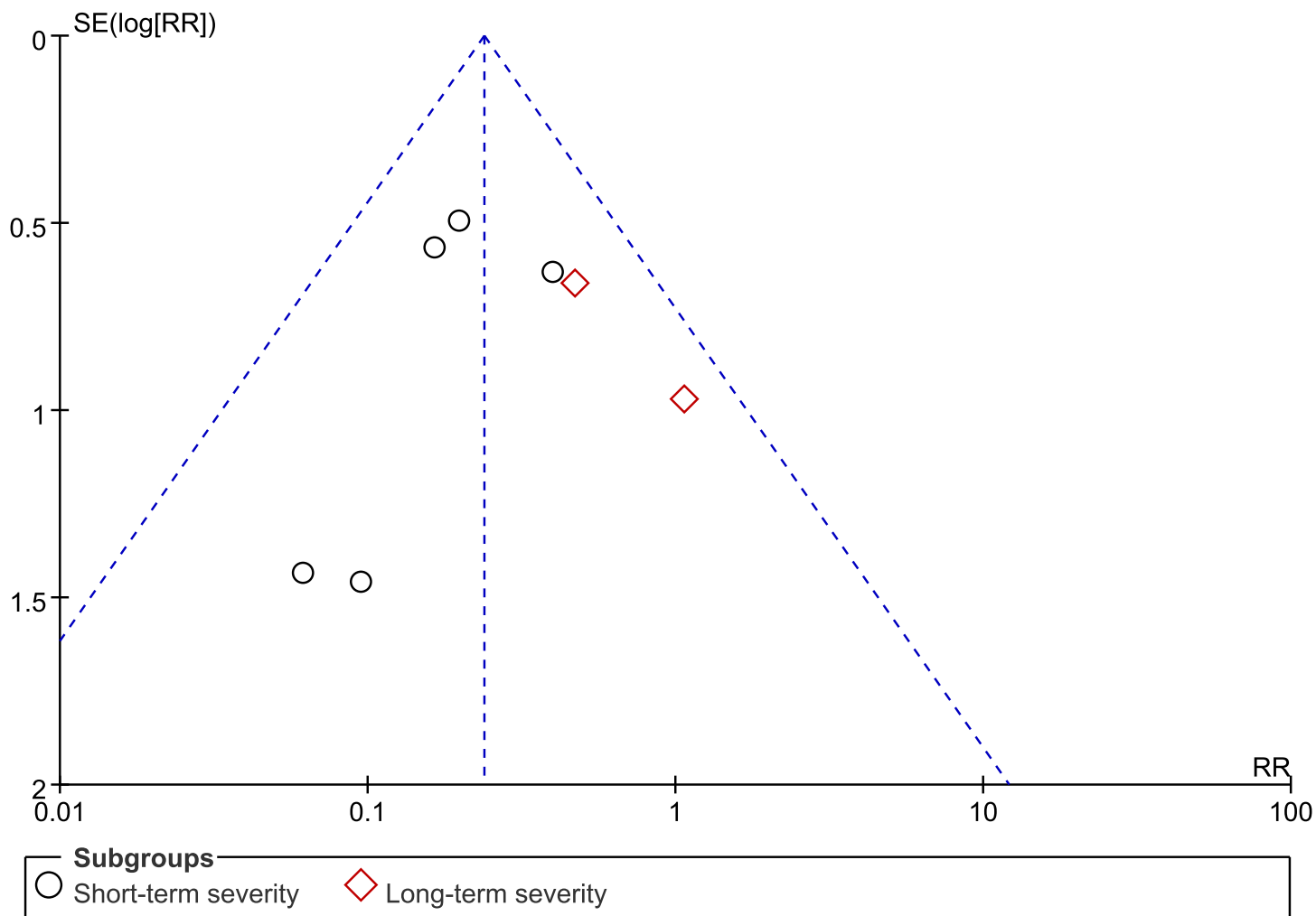

Supplement: Supplementary file 1 [file DataSheet2.PDF]

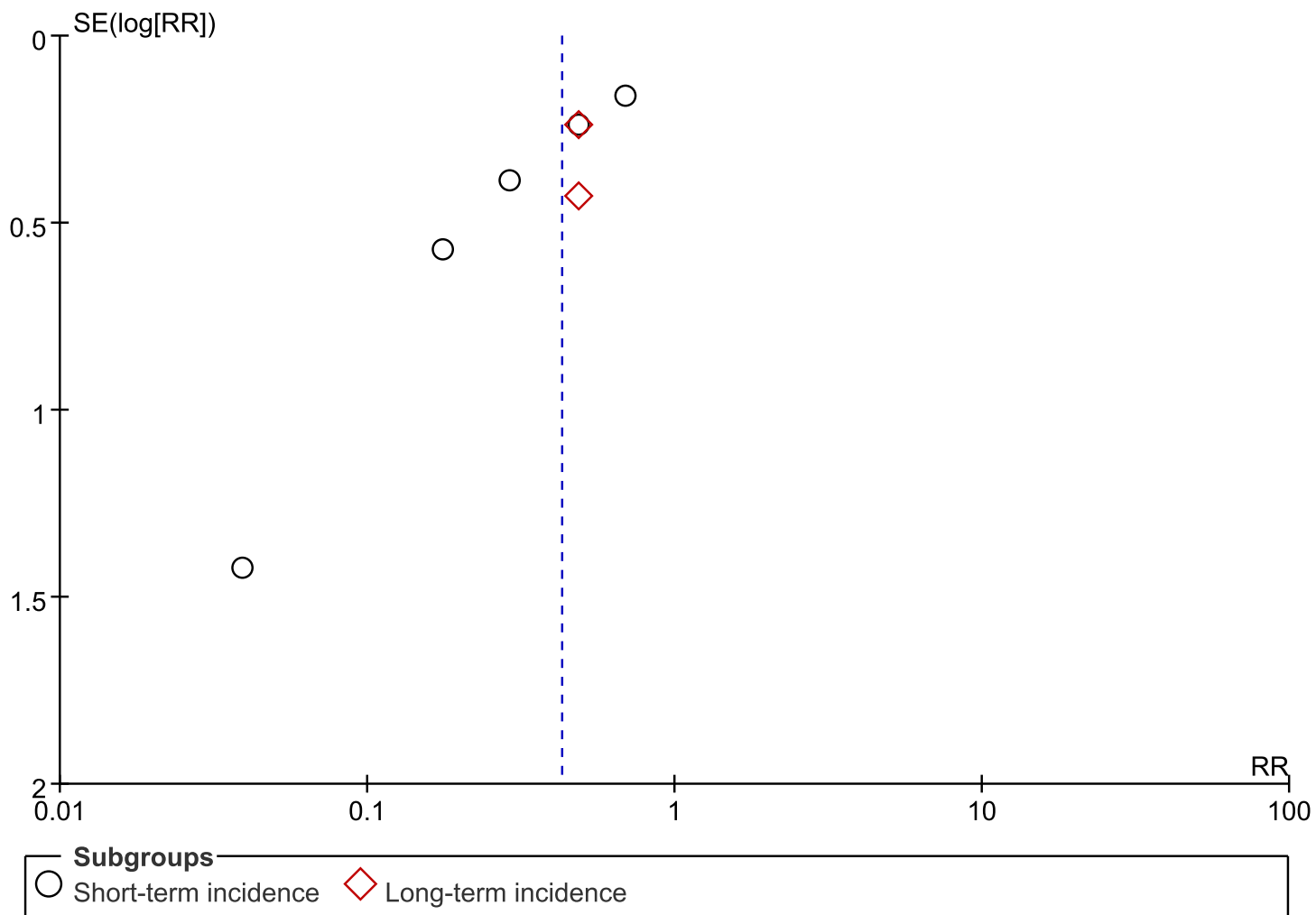

Supplement: Supplementary file 2 [file DataSheet3.PDF]

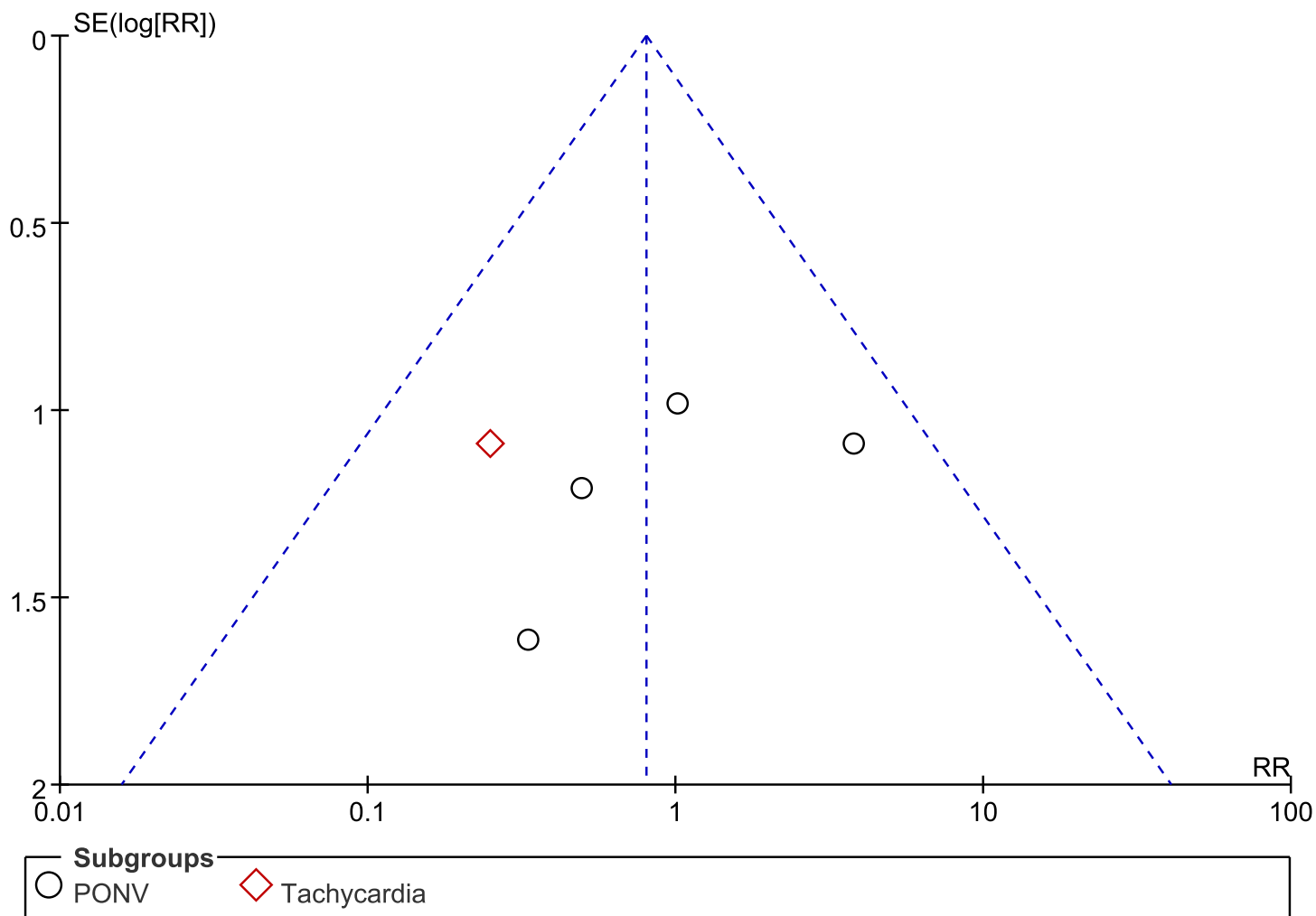

Supplement: Supplementary file 3 [file DataSheet1.PDF]
